# SF-1 Induces Nuclear PIP2

**DOI:** 10.3390/biom13101509

**Published:** 2023-10-12

**Authors:** Ethan S. Chi, Elizabeth A. Stivison, Raymond D. Blind

**Affiliations:** Department of Medicine, Division of Diabetes, Endocrinology and Metabolism, Vanderbilt University Medical Center, Nashville, TN 37232, USA

**Keywords:** Ad4BP, NR5A, Inositol polyphosphate multikinase IPMK, non-membrane nuclear lipids

## Abstract

Metazoan cell nuclei contain non-membrane pools of the phosphoinositide lipid PI(4,5)P2 (PIP2), but how this hydrophobic lipid exists within the aqueous nucleoplasm remains unclear. Steroidogenic Factor-1 (NR5A1, SF-1) is a nuclear receptor that binds PIP2 in vitro, and a co-crystal structure of the complex suggests the acyl chains of PIP2 are hidden in the hydrophobic core of the SF-1 protein while the PIP2 headgroup is solvent-exposed. This binding mode explains how SF-1 can solubilize nuclear PIP2; however, cellular evidence that SF-1 expression associates with nuclear PIP2 has been lacking. Here, we examined if tetracycline induction of SF-1 expression would associate with nuclear accumulation of PIP2, using antibodies directed against the PIP2 headgroup. Indeed, tetracycline induction of wild-type SF-1 induced a signal in the nucleus of HEK cells that cross-reacts with PIP2 antibodies, but did not cross-react with antibodies against the lower abundance phosphoinositide PI(3,4,5)P3 (PIP3). The nuclear PIP2 signal co-localized with FLAG-tagged SF-1 in the nuclear compartment. To determine if the nuclear PIP2 signal was dependent on the ability of SF-1 to bind PIP2, we examined a “pocket mutant” of SF-1 (A270W, L345F) shown to be deficient in phospholipid binding by mass spectrometry. Tetracycline induction of this pocket mutant SF-1 in HEK cells failed to induce a detectable PIP2 antibody cross-reactive signal, despite similar Tet-induced expression levels of the wild-type and pocket mutant SF-1 proteins in these cells. Together, these data are the first to suggest that expression of SF-1 induces a PIP2 antibody cross-reactive signal in the nucleus, consistent with X-ray crystallographic and biochemical evidence suggesting SF-1 binds PIP2 in human cells.

## 1. Introduction

Over 35 years ago, Lucio Cocco working with Robin Irvine generated some of the first data suggesting that detergent-resistant intra-nuclear pools of PI(4,5)P2 (PIP2) were regulated during the differentiation of mammalian cells [1]. Over the following several decades, work from Peter Downes [2], Nullin Divecha [3], Pavel Hozak [4], and Richard Anderson [5] among others [6,7,8,9] who reviewed in this special issue [10], suggested the presence of phosphoinositides in non-membrane compartments within the nucleoplasm. Several proteins in the nucleus have been suggested to interact with nuclear phosphoinositides including MPRIP [11], TAF3 [12], ING2 [9], Nucleophosmin/B23 [13], STAR-pap [14], and BAF [8,15]; however, no detailed structural biology on how these proteins bind to phosphoinositides is available. One clear example, complete with structural details at atomic resolution explaining how nuclear PI(4,5)P2 can exist in non-membrane pools in the nucleoplasm [16], is the nuclear receptor Steroidogenic Factor-1 (*NR5A1*, SF-1). The X-ray crystal structure of PI(4,5)P2 bound to the SF-1 ligand-binding domain shows the hydrophobic acyl chains of PI(4,5)P2 are hidden deep in the hydrophobic core of the SF-1 protein, while the hydrophilic phosphoinositide headgroup is solvent-exposed [16]. This structure provides a clear mechanism that can explain the apparent membrane-independent existence of nuclear PI(4,5)P2, yet no single physicochemical explanation of how PI(4,5)P2 exists in non-membrane compartments is consistent with all the data published thus far, including SF-1 [17]. Although the endogenous ligand for SF-1 has not been conclusively identified in mammalian cells, bacterial phospholipids co-purify and co-crystalize with SF-1 from recombinant *E. coli* expression systems [18], and several phospholipids present in mammalian cells have been co-crystalized with SF-1, including phosphatidylcholine [19], PI(4,5)P2 [16] and the far less abundant phosphoinositide PI(3,4,5)P3 (PIP3) [16].

SF-1 is a member of the nuclear receptor superfamily of ligand-activated transcription factors [20,21,22,23], and is only expressed in the gonads, adrenals, and the ventral-medial region of the hypothalamus [24,25] with important physiological functions in steroidogenesis, sexual development, and estrogen physiology [26,27,28,29,30,31]. Global loss of SF-1 in mice is perinatally lethal due to adrenal agenesis, which can be rescued by exogenous corticosteroids [32,33,34]. Like many nuclear receptors, SF-1 is a potential drug target in several human diseases, including the rare cancer adrenocortical carcinoma [35,36], and endometriosis, which affects about half of all women [26,37,38,39]. The full-length SF-1 protein consists of an N-terminal DNA-binding domain connected to the C-terminal phospholipid ligand-binding domain (LBD) via an unstructured hinge domain [40]. Although the three-dimensional structure of the full-length SF-1 remains undetermined [41], several structures of the phospholipid ligand-binding domain have been solved, including three crystal structures bound to different phosphoinositides [42], including one crystal structure of PI(4,5)P2 bound to SF-1 [16].

Several lines of evidence suggest PI(4,5)P2 is an endogenous, regulatory ligand for SF-1. The nuclear inositol polyphosphate multi-kinase (IPMK) can directly phosphorylate PI(4,5)P2 bound to SF-1 with about sixfold better kinetic parameters (kcat/K_M_ = 520,000 s^−1^M^−1^) than IPMK phosphorylation of PI(4,5)P2 in micelles (kcat/K_M_ = 82,000 s^−1^M^−1^). Chemical or genetic downregulation of IPMK activity regulates SF-1 transcriptional output in human cells, but the same downregulation of IPMK has no effect on a “pocket mutant” of SF-1 (A270W, L345F) that does not bind PI(4,5)P2 [43]. The PI(4,5)P2-generating phosphatase PTEN has robust phosphatase activity on PI(3,4,5)P3 bound to SF-1 to generate PI(4,5)P2 bound to SF-1 (V_MAX_ = 0.7 ± 0.1 µmol/min/mg; kcat = 0.59 ± 0.1 s^−1^; K_M_ = 1.0 ± 0.7 µM; kcat/K_M_ = 591,000 s^−1^M^−1^) and overexpression of PTEN in PTEN-null cells downregulated SF-1 activity [43]. The X-ray crystal structures of SF-1 bound to PIP2 and PIP3 suggest the phosphoinositide headgroups create an interaction surface for coregulator proteins [16]. Finally, when SF-1 is immunoprecipitated from human HEK cells, ^32^P-ATP radiolabel is incorporated into these immunoprecipitates by the kinase IPMK using in vitro kinase assays, but immunoprecipitates of the “pocket mutant” of SF-1 (A270W, L345F) that does not bind PI(4,5)P2 fail to incorporate radiolabel [43]. Further, the radiolabel from ^32^P-ATP does not incorporate into any protein component of these immunoprecipitates, and the radiolabel is non-covalently associated as it can be competed away with unlabeled SF-1 ligands [43]. Thus, the data are most consistent with PIP2 directly binding SF-1 in mammalian cells. However, all these approaches have relied on the biochemical purification of SF-1 from cells. Thus, it remains formally possible that SF-1 interacts with PI(4,5)P2 only once the cells have been broken open during the purification of SF-1 protein. No study has demonstrated an association between SF-1 and PI(4,5)P2 in fixed cells where SF-1 has not been biochemically purified.

Indeed, despite the structural biology and functional evidence linking PI(4,5)P2 to SF-1, surprisingly few studies have attempted to confirm that PI(4,5)P2 associates with SF-1 in mammalian cells. One study used a PH domain biosensor to co-localize PIP3 with SF-1 in UV-induced fluorescence recovery after bleaching (FRAP) experiments [44]. Similarly, a discovery-based mass spectrometry study by Marion Sewer and Al Merrill identified sphingolipids associated with SF-1 in a human adrenal cell line, these sphingolipids had decreased association with several mutants of SF-1 [45,46]; however, PI(4,5)P2 again was not tested for. Phosphoinositides are difficult to detect in discovery-based mass spectrometry as they require highly specialized protocols to be detected [47,48]. Thus, although much data suggests PIP2 associates with biochemically purified SF-1, direct evidence that SF-1 expression might induce PI(4,5)P2 accumulation in the nucleus has been lacking.

Here, we begin to address this gap by examining the nuclear accumulation of the immunofluorescence signal from antibodies directed against the headgroup of PI(4,5)P2, after genetic induction of SF-1 in human HEK cells. We find that when SF-1 is expressed in HEK cells bearing a single, isogenic, stably integrated tetracycline-inducible copy of wild-type SF-1, a PI(4,5)P2 antibody cross-reactive immunofluorescent signal increases within the nucleoplasm. However, induction of this same nuclear PIP2 antibody cross-reactive signal cannot be detected upon identical induction of the SF-1 pocket mutant (A270W, L345F) in HEK cells; a mutant previously established to lack detectable binding to any phospholipids, including PI(4,5)P2, despite equal levels of wild-type vs. pocket mutant SF-1 protein expression [43]. Together, the data presented here support previous structural [16] and functional [43,44] models of SF-1, which together suggest SF-1 binds nuclear PI(4,5)P2 in human cells. These data are the first to demonstrate an association between SF-1 and nuclear PI(4,5)P2 in fixed human cells.

## 2. Methods

### 2.1. Materials

Dulbecco’s modified Eagle’s medium (DMEM) was from Gibco (Grand Island, NY, USA), tetracycline-tested fetal bovine serum was from Bio-Techne (Minneapolis, MN, USA), chamber slides and all other standard cell culture reagents were purchased from Fisher Scientific (Waltham, MA, USA). Tetracycline was purchased from Gibco (Grand Island, NY, USA), nocodazole was purchased from Millipore Sigma (Burlington, MA, USA), and glycine was from Fisher (Hampton, NH, USA). Phosphate buffered saline was from Gibco BRL (Grand Island, NY, USA). Triton X-100 and TWEEN20 were purchased from Sigma (St. Louis, MO, USA). DAPI nuclear stain was Invitrogen (Waltham, MA, USA).

### 2.2. Antibodies

The 2C11 affinity purified anti-PI(4,5)P2 antibody was from Echelon (Salt Lake City, UT, USA) product number Z-P045, lot number XCM042523-23, and used at 1:200 dilution in blocking buffer (see IF methods for blocking buffer). The affinity-purified anti-PI(3,4,5)P3 antibody was also from Echelon product number Z-G345, lot number ML120516-23, and used at 1:200 dilution in blocking buffer. The anti-3X FLAG M2 monoclonal antibody was from Sigma (St. Louis) and used at 1:1000 dilution in blocking buffer. For colocalization of FLAG and PIP2 in the same chamber, mouse FLAG-primary M2 antibodies were detected with 1:1000 PBS-diluted Invitrogen Alexa-Fluor 594 goat anti-mouse-IgG1, lot number 2566384, while 2C11 anti-PIP2 primary antibodies were detected with 1:2000 PBS-diluted Invitrogen Alexa-Fluor 488 goat anti-mouse-IgM, lot number 1896382. The anti-actin antibody was from Cell-Signaling (product number 8H10D10) and was used at 1:5000 dilution in 1X TBST. The secondary antibody in the Westerns was Promega anti-mouse HRP conjugate (product number W402B) and was used at 1:10,000 dilution in 1X TBST.

### 2.3. SF-1 Induction

The previously published Tetracycline-inducible HEK cell line was constructed using the using the Invitrogen Flp-In T-REx system [43]. The cell line contains a stable, isogenic tetracycline-inducible copy of N-terminally 3X FLAG-tagged wild-type (WT) or “pocket mutant” (A270W, L345F) mouse SF-1. These cells were grown at 37 °C in 5% CO_2_ in standard Dulbecco’s modified Eagle’s medium (DMEM) supplemented with 10% tetracycline-tested fetal bovine serum, 15 ug/mL blasticidin (Thermo) to maintain the SF-1 cassette, and 100 ug/mL hygromycin (Thermo) to maintain the Tet-repressor cassette. Four-well chamber slides were seeded with 60,000 cells per chamber in 1 mL of the above media; after cells reached 50–60% confluency, SF-1 expression was induced with 100 ng/mL tetracycline or an equal volume of 100% ethanol vehicle control for a total of 24 h at 37 °C in 5% CO_2_ in the same cell culture incubator. These cells were then processed for chamber slide fixation and permeabilization below.

### 2.4. Induction of Nuclear PI(4,5)P2 Cross-Reactive Signal by Serum Starvation

Indicated cells were grown at 37 °C in 5% CO_2_ in DMEM supplemented with 10% tetracycline-tested fetal bovine serum, four-well chamber slides were seeded with 60,000 cells per chamber in 1 mL of media; after cells reached 50–60% confluency, the media over the cells were changed to either control DMEM with 10% fetal bovine serum or identical DMEM lacking serum. Cells were incubated in these two conditions for 24 h at 37 °C in 5% CO_2_ in the same cell culture incubator; the cells were then processed for chamber slide fixation and permeabilization below.

### 2.5. Chamber Slide Fixation and Permeabilization

Tetracycline (100 ng/mL) or ethanol vehicle-induced cells were fixed by adding 4% formaldehyde (Sigma, 16% single-use ampule) to the media, incubating at room temperature for 10 min, and quenched with 620 mM glycine for 5 min at room temperature. Cells were then gently washed 3× with 0.2 µm filtered ice-cold phosphate-buffered saline and membranes permeabilized in each chamber with 500 µL of 0.1% Triton X-100 for 10 min at room temperature; the cells were then washed another three times with ice-cold 1X PBS. Permeabilized cells were then blocked in 3% bovine serum albumin (RPI) and 0.5% Tween in PBS overnight at 4 °C.

### 2.6. Immunofluorescence

To prepare for primary antibody incubation, cells in chamber slides blocked as detailed above were gently washed three times with ice-cold 1X PBS. 3XFLAG-SF-1 nuclear localization was verified using Sigma M2 anti-3X FLAG antibodies diluted 1:1000 in 1X PBS and allowed to probe overnight at 4 °C. Antibodies directed against PI(4,5)P2 were diluted 1:200 in 1X PBS and probed overnight at 4 °C. All four chambers for each slide were used for only a single primary antibody, and a single chemical treatment (Ethanol vehicle, tetracycline treatment), so no cross-chamber contamination was possible. When only one primary antibody was used, both anti-3X FLAG and anti-PI(4,5)P2 primary antibody probed slides were then exposed to the same anti-mouse secondary antibodies (594 nm Alexa-Fluor, Cell Signaling Technologies product number 8890S) diluted 1:500 in 1X PBS and incubated in each chamber for one hour at room temperature in the dark. For FLAG co-localization studies, 1:1000 PBS-diluted anti-mouse-IgG1-specific secondary antibodies (Invitrogen Alexa-Fluor 488 goat anti-mouse-IgM, lot number 1896382) were used to detect the Sigma M2 anti-FLAG primary antibodies; these secondary antibodies were confirmed by IF to have undetectable cross-reactivity with the 2C11 anti-PIP2 IgM-class antibodies. In the same chamber for anti-PIP2 co-localization studies, 1:2000 PBS-diluted anti-mouse-IgM-specific secondary antibodies (Invitrogen Alexa-Fluor 594 goat anti-mouse-IgG1, lot number 2566384) were used to detect the 2C11 anti-PIP2 primary antibodies; again, these secondary antibodies were also confirmed by IF to have undetectable cross-reactivity with Sigma M2 anti-FLAG antibodies. Following secondary antibody incubation, the cells were washed three times with ice-cold 1X PBS and the chambers were removed. ProLong Gold (Invitrogen) containing DAPI DNA stain was used as mounting medium and a coverslip was added. All immunofluorescence images were acquired using an EVOS FLoid Cell Imaging System. Brightness of all images in each figure was equally increased as indicated in each figure legend, no other adjustments to the images were made after acquisition at the microscope, all images were acquired at the microscope under identical settings, all replicate images are provided as supplemental data, and all original TIF files are available upon request.

### 2.7. Image Quantitation

Cell counts were determined using Image J software Version 1.53t [49]. DAPI images were converted to binary images and the watershed function in ImageJ was used to separate any overlapping cells. The total number of cells was determined using particle analysis in ImageJ with thresholds set from 120 to infinity. The resulting DAPI/nuclear outlines were overlaid onto the PIP2-stained IF images and manually counted to arrive at the number of nuclei with PIP2 staining in each field. For each experiment, at least 3 fields were quantitated in this way, and each field chosen for quantitation was representative of its respective chamber.

### 2.8. Western Blotting

Tetracycline (100 ng/mL) or ethanol vehicle-induced cells were harvested in RIPA buffer with Roche protease inhibitors (without EDTA). Cells were scraped into a chilled microfuge tube and centrifuged at 13,000 rpm in a microcentrifuge for 15 min at 4C. The supernatant was run on a 10% Bis-Tris SDS-PAGE gel at 140 V. The proteins were transferred to a 0.2 µm nitrocellulose membrane in 1X Novex NuPage transfer buffer (Carlsbad, CA, USA) at 10 V for one hour. The membrane was blocked with 10% milk in 1X TBST for one hour at room temperature. The membrane was washed twice with 1X TBST for five minutes at room temperature and probed with 1:1000 diluted Sigma M2 anti-3X FLAG antibodies in 1X TBST for 1 h at room temperature. The membrane was washed another two times with 1X TBST and incubated with 1:10,000 diluted HRP secondary antibody (Promega, product number W402B) in 1X TBST at room temperature for one hour. The same procedure and antibody dilutions were used with mouse anti-beta actin antibodies (Cell-Signaling product number 8H10D10). Images of Western blots were acquired on a BioRad ChemiDoc imaging system.

## 3. Results

### 3.1. Ectopic Expression of SF-1 Induces an Immunofluorescence Signal in the Nucleus of HEK293 Cells That Is Cross-Reactive with PI(4,5)P2 Antibodies

**Figure 1:**
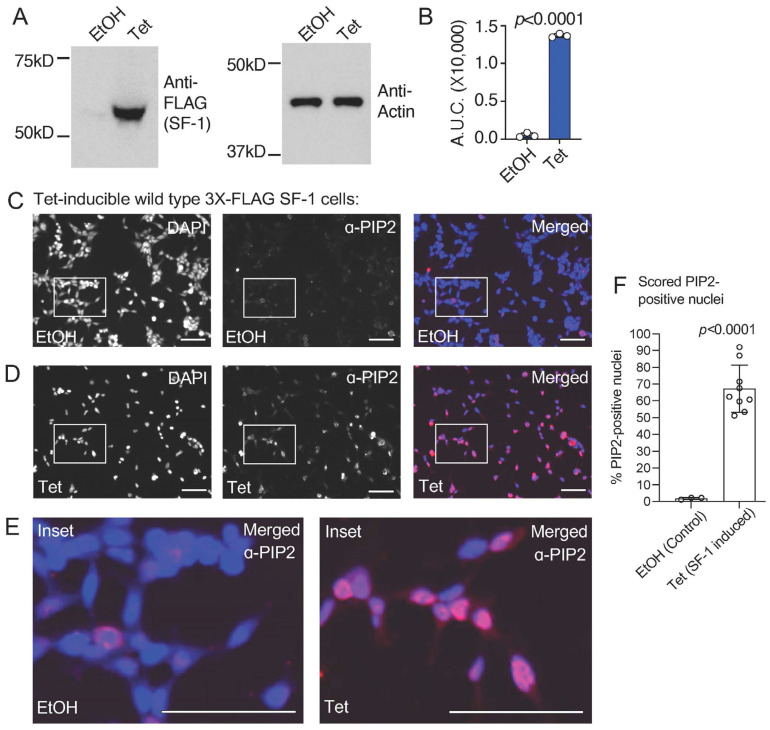
Tetracycline-induced expression of wild type SF-1 in HEK stable cells induces a nuclear signal cross reactive with PI(4,5)P2 (PIP2) antibodies. (**A**) Western blots showing 3X-FLAG wild type SF-1induction by tetracycline and anti-actin loading control, (**B**) westerns were quantified and area under the curves analyzed by unpaired *t*-test, showing relative induction of SF-1 protein, error is standard deviation. (**C**) 40× immunofluorescence images of HEK cells bearing a stably-integrated, isogenic copy of3X-FLAG tagged wild-type SF-1, treated for 24 h with ethanol vehicle control, left is DAPI nuclear/DNA stain (blue), middle is PI(4,5)P2 (PIP2) antibody staining (red) and right is merged (DAPI and anti-PIP2), bar = 125 µm in all panels. (**D**) Identical as in (**A**) but cells treated 24 h with 100 ng/mL tetracycline to induce wild-type SF-1 expression (confirmed by western). (**E**) Magnified insets of ethanol vehicle control (left) or tetracycline treated (right) insets from above immunofluorescence images. (**F**) Cell nuclei were counted using Image J and nuclear PIP2 antibody staining manually counted (see methods), analyzed by unpaired *t*-test. All images had brightness identically increased by 50%, DAPI (blue) and anti-PIP2 (red) images alone were color desaturated. These data suggest tetracycline induction of SF-1 associates with a nuclear signal that cross-reacts with antibodies directed against the Pl(4,5)P2 headgroup. Original images can be found in Figure S2.

**Figure 2:**
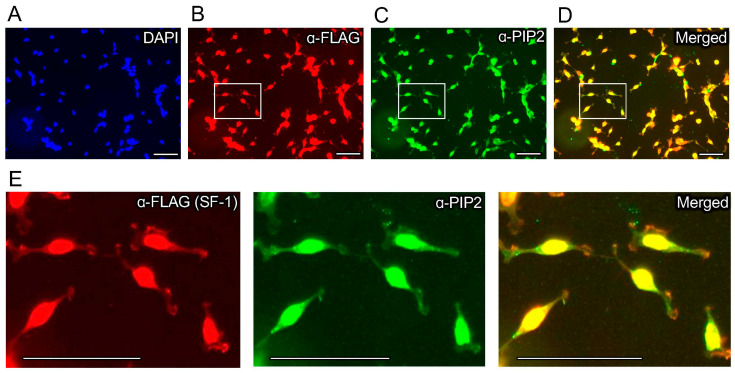
Immunofluorescence co-staining with anti-FLAG and anti-PIP2 antibodies co-localizes to the nucleus of HEK cells. Top row are 40× immunofluorescence images of HEK cells bearing a stably-integrated copy of wild-type 3X-FLAG-SF-1, treated 24 h with 100 ng/mL tetracycline to induce SF-1 expression, showing (**A**) DAPI staining (blue), (**B**) anti-3XFLAG antibody staining (red) to visualize 3X-FLAGtagged SF-1, (**C**) anti-PIP2 antibody staining (green) and (**D**) the merged anti-FLAG (red, SF-1) with anti-PIP2 (green) staining, bar = 125 µm in all panels. (**E**) Magnified indicated insets from white boxes of anti-FLAG (left), anti-PIP2 (middle) and merged (right) images from above, all images in all panels had brightness increased identically by 50% and are representative of 3 independent experiments, secondary antibodies were confirmed to be specific for anti-FLAG (Sigma M2) primary antibodies (lgG1-specificsecondaries) and anti-PIP2 (2C11) primary antibodies (lgM-specific secondaries), see methods for details. These data suggest anti-FLAG-SF-1 signal co-localizes with the signal from PIP2 antibodies, in these cells by immunofluorescence.

We previously established an isogenic HEK cell line with a single, stably integrated tetracycline-inducible 3X-FLAG-tagged wild-type SF-1, characterizing the induction, expression, and gene regulatory activity of SF-1 in these cells [43]. We used these cells in standard formaldehyde-fixed immunofluorescence (IF) studies with commercially available PI(4,5)P2 (PIP2) monoclonal antibodies. We first confirmed the tetracycline-induction of wild-type SF-1 protein over the ethanol vehicle control by Western blot (Figure 1A,B) and immunofluorescence (Supplemental Figure S1), which has been previously shown in another published study [43]. Immunofluorescence staining using these PIP2 antibodies in the uninduced, ethanol vehicle control-treated cells suggests a relatively low level of membrane staining as expected for PI(4,5)P2 (Figure 1C). However, the induction of wild-type SF-1 by the addition of tetracycline to the media for 24 h induced robust nuclear staining, cross-reactive with the anti-PI(4,5)P2 antibodies (Figure 1D, Supplemental Figure S2), which was also observed when representative regions were magnified to compare the ethanol vehicle to tetracycline-treated cells (Figure 1E). We counted the number of nuclei with nuclear PI(4,5)P2 signal (see Methods), which showed a significant increase in PI(4,5)P2-antibody staining positive nuclei in the tetracycline-induced wild-type SF-1 cells compared to the ethanol control (Figure 1F). We next asked if the signals from the anti-FLAG (SF-1) and anti-PIP2 antibodies would co-localize in the same cells by immunofluorescence. Indeed, the anti-PIP2 signal co-localized with SF-1 in the nucleus of these HEK cells upon tetracycline induction (Figure 2A–E). These data suggest that the tetracycline-induced expression of wild-type SF-1 results in the significant accumulation of an immunofluorescence signal in the nucleus of HEK cells, which cross-reacts with PI(4,5)P2 antibodies.

### 3.2. The Signal Induced by Expression of SF-1 in HEK Cells Does Not Cross-React with PI(3,4,5)P3 Antibodies

**Figure 3:**
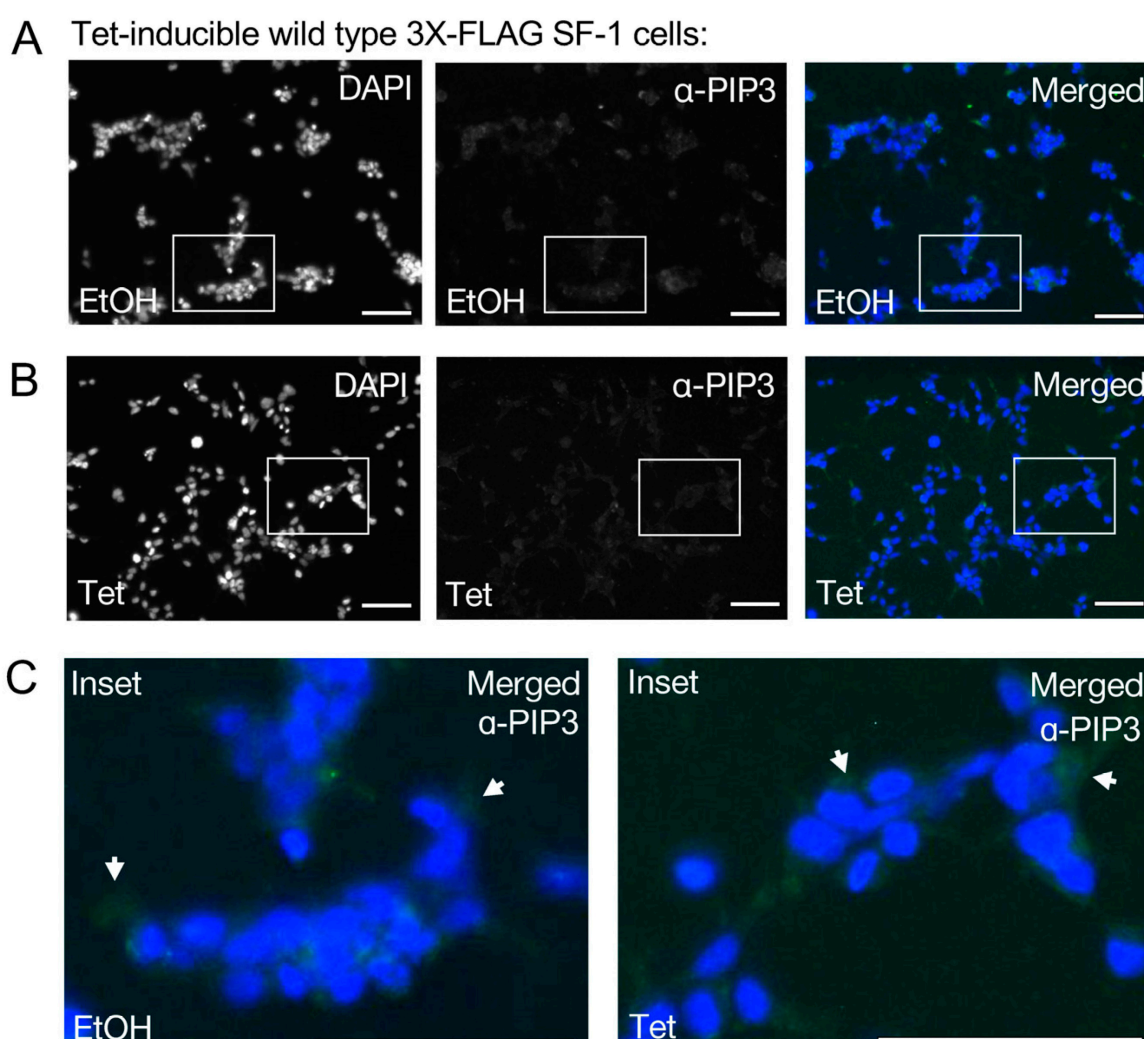
Antibodies against Pl(3,4,5)P3 (PIP3) do not detect an inducible signal upon tetracycline-induced expression of wild type SF-1in HEK cells. (**A**) 40× immunofluorescence images of HEK cells bearing a stably-integrated copy of wild-type SF-1, treated 24 h with ethanol control, panel as indicated DAPI (blue), PIP3 antibody (green), merged (DAPI and anti-PIP3), bar = 125 µm in all panels. (**B**) Same as (**A**) but cells but treated 24 h with 100 ng/mL tetracycline to induce wild-type SF-1. (**C**) Magnified insets of ethanol vehicle (left) or tetracycline treated (right) from merged images, arrows show slight green staining with PIP3 antibodies (not in the nucleus), all images in all panels had brightness increased identically by 50%, DAPI (blue) and anti-PIP3 (green) images were color desaturated. These data suggest tetracycline induction of SF-1 does not result in detectable signal that cross-reacts with PIP3 antibodies, under the conditions of this assay.

We next asked if the nuclear signal induced by the tetracycline induction of wild-type SF-1 would also cross-react with PI(3,4,5)P3 (PIP3) antibodies (Figure 3A). The tetracycline induction of wild-type SF-1 was unable to induce a detectable signal cross-reactive with PIP3 antibodies under the same conditions tested with PIP2 antibodies (Figure 3B, Supplemental Figure S3). Representative regions were magnified from merged images to compare PIP3 antibody staining between ethanol and tetracycline-treated cells, also suggesting no detectable signal was induced (Figure 3C). However, we are quick to note that PI(3,4,5)P3 is present at lower overall levels in cells than PI(4,5)P2, the low levels of PI(3,4,5)P3 that are produced are usually more transient than PI(4,5)P2 levels, and, lastly, these cells were not specifically induced to produce any excess PI(3,4,5)P3 that may enhance detection. These data suggest that the PIP2 antibody cross-reactive signal observed upon induction of wild-type SF-1 does not detectably cross-react with antibodies directed against PIP3, under the conditions of this assay.

### 3.3. A Mutant of SF-1 Deficient in Phospholipid Binding Induces Less Signal Cross-Reactive with PI(4,5)P2 Antibodies in HEK Cell Nuclei

**Figure 4:**
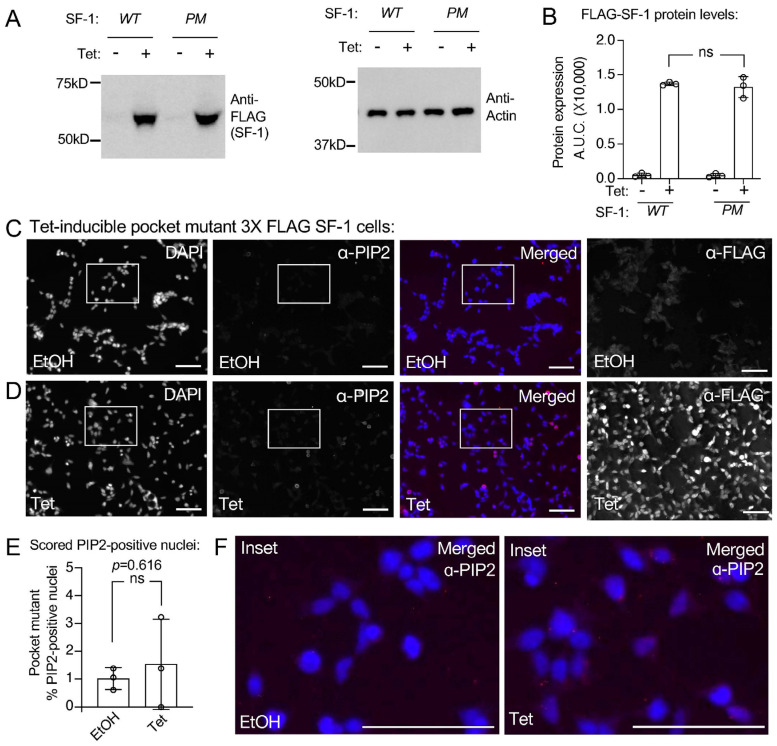
The pocket mutant of SF-1 does not induce a signal cross reactive with PIP2 antibodies. (**A**) Western blot showing SF-1 induction for 24 h with 100 ng/mL tetracycline of wild type vs. pocket mutant SF-1 with anti-actin loading control and (**B**) quantitation of the westerns analyzed by unpaired *t*-test, error is standard deviation. (**C**) 40× IF images of HEK cells bearing a stably-integrated copy of the pocket mutant SF-1 (A270W, L345F), previously shown to not bind phospholipids including PIP2. Cells were treated for 24 h with ethanol vehicle, IF staining as indicated, DAPI (blue), PIP2 antibody (red) merged DAPI and PIP2, anti-FLAG antibody (SF-1, red), bar = 125 µm in all panels. (**D**) Same as (**C**) but cells treated 24 h with 100 ng/mL tetracycline to induce pocket mutant SF-1. (**E**) Percentage of pocket mutant SF-1 cell nuclei scored positive for nuclear PIP2 antibody staining (see methods), error is standard deviation. (**F**) Magnified insets of merged images from ethanol (left) or tetracycline treated (right) insets, DAPI (blue) and PIP2 antibody staining (red). All images had brightness increased identically to 50%, DAPI and anti-PIP2 image color was desaturated. These data suggest tetracycline induction of the pocket mutant of SF-1 does not accumulate a PIP2 antibody cross reactive signal. Original images can be found in Figure S4.

In addition to establishing tetracycline-inducible HEK cell lines expressing wild-type SF-1, we also established isogenic HEK cell lines that express a two-amino acid mutant of SF-1 (A270W, L345F). This SF-1 “pocket mutant” [18,19,43] is deficient in binding to all phospholipids by mass spectrometry [18], and when this mutant was purified from tetracycline-inducible HEK cell lines, it was also shown to be devoid of PI(4,5)P2 in biochemical assays [43]. Although the pocket mutant is hypomorphic, it retains about half the wild-type SF-1 function [18]. Using the pocket mutant SF-1 HEK cell line, we confirmed similar expression levels of the wild-type vs. pocket mutant SF-1 upon identical 100 ng/mL tetracycline induction for 24 h by Western blot (Figure 4A,B) and immunofluorescence (Figure 4C,D). These data are consistent with previously published Westerns and IF that also show no change in the expression levels of the pocket mutant SF-1 (A270W, L345F) compared to the wild-type [43]. Immunofluorescence with PIP2 antibodies after the cells had been treated 24 h with the ethanol vehicle (Figure 4C) or 100 ng/mL tetracycline to induce the pocket mutant SF-1 (Figure 4D, Supplemental Figure S4) had no detectable induction of the nuclear signal cross-reactive with PIP2 antibodies (Figure 4E,F). These data suggest that the full induction of the PIP2 antibody cross-reactive signal is dependent upon the ability of SF-1 to bind phospholipids.

### 3.4. Direct Comparison of Wild-Type to Pocket Mutant SF-1 Shows Wild-Type SF-1 Induces Significantly More Nuclear PIP2 Antibody Cross-Reactive Signal

**Figure 5:**
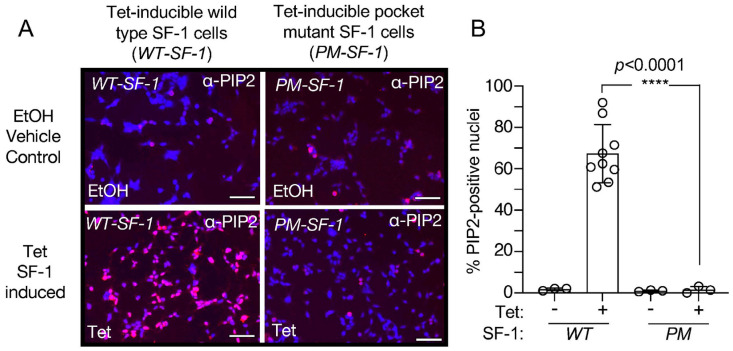
Representative merged IF images (DAPI merged with PIP2-antibody stained) to directly compare wild type vs. pocket mutant SF-1 (A270W, L345F). (**A**) Merged DAPI (blue) and anti-PIP2 staining (red) of wild type (left) vs. pocket mutant (right) SF-1 cells with indicated treatments of EtOH (top) or 100 ng/mL tetracycline (bottom). For clarity, these images are identical to those in Supplemental Figures S1 and S3, except with image brightness increased identically in all panels by 60% and placed side by side for direct comparison. Scale bar represents 125 µm, images all acquired at 40× under identical settings. (**B**) Quantitation of the percentage of PIP2-positive nuclei in IF images, comparing tetracycline induced wild type SF-1 to tetracycline induced pocket mutant SF-1 by unpaired *t*-test, all error is standard deviation. These data suggest tetracycline-induced wild-type SF-1 cells have significantly more nuclear accumulation of PIP2-antibody staining than identical tetracycline induction of the pocket-mutant of SF-1.

We then directly compared the tetracycline-induced immunofluorescence nuclear signals from the PIP2 antibodies between wild-type and pocket mutant SF-1 cell lines (Figure 5A, Supplemental Figure S5). An unpaired *t*-test comparing the percentage of PIP2-positive nuclei shows wild-type SF-1 induces significantly more nuclear PIP2 antibody cross-reactive signal than the pocket mutant of SF-1 (*p* < 0.0001, Figure 5B), suggesting that the nuclear PIP2 antibody signal is only induced by tetracycline in the wild-type SF-1 cell line, despite both the pocket mutant and wild-type SF-1 cell lines expressing SF-1 within the nucleus [43]. These data suggest that the tetracycline induction of the phospholipid-binding deficient pocket mutant SF-1 (A270W, L345F) results in the undetectable accumulation of the nuclear PIP2 antibody cross-reactive signal, when compared to the tetracycline induction of the wild-type SF-1. Together, these data suggest that induction of wild-type SF-1 associates with nuclear accumulation of a signal that is cross-reactive with PIP2 antibodies.

## 4. Discussion

The data presented here suggest that the ectopic expression of SF-1 in HEK cells associates with the induction of a nuclear signal that cross-reacts with PI(4,5)P2 (PIP2) antibodies. Full induction of this signal appears to require the ability of SF-1 to bind phospholipids, as a mutant of SF-1 that is deficient in binding PIP2 does not induce a detectable signal by immunofluorescence, despite the mutant protein being expressed equally as wild-type SF-1, and within the nuclear compartment. Thus, the induction of the nuclear PIP2 signal cannot be explained by the tetracycline used to induce SF-1, as the pocket mutant SF-1 was induced by an identical tetracycline treatment. Further, the signal did not cross-react with PI(3,4,5)P3 (PIP3) antibodies, suggesting a degree of specificity; however, basal PI(3,4,5)P3 levels are much lower than PI(4,5)P2 levels so the lack of a PI(3,4,5)P3 signal may simply reflect less PI(3,4,5)P3 in the cells. Although other studies have used structural biology [16] and functional experiments [43] to connect PI(4,5)P2 to SF-1, this study presents the first evidence suggesting that the expression of SF-1 induces a nuclear PI(4,5)P2 signal in human cells. What this study cannot formally address is if the PIP2 antibody is directly recognizing PIP2 bound to SF-1, or if the signal is an undiscovered, indirect byproduct of SF-1 expression, e.g., SF-1 induction of a downstream product that produces the nuclear PIP2 signal, but which is only induced by wild-type and not pocket mutant SF-1, and which does not cross-react with PIP3 antibodies. Although that possibility cannot be formally excluded, the simplest interpretation of the data presented here is that the nuclear signal from the PIP2 antibodies is recognizing PIP2 bound to SF-1, and the increase in total PIP2 signal results from compensation for PIP2 extracted from cellular membranes by SF-1. Although highly speculative, this hypothesis is also supported by our observation that the nuclear PIP2 signal is not detectable when the pocket mutant of SF-1 is induced in these assays. The pocket mutant of SF-1 (A270W, L345F) [18,43] has been validated by mass spectrometry to lack co-purifying phospholipids [18]. Since the pocket mutant does not bind PIP2, it would not increase the nuclear levels of PIP2, despite the pocket mutant protein being expressed to equal levels as the wild-type (Figure 4B).

There is considerable evidence suggesting phospholipids other than PI(4,5)P2 bind directly to SF-1. Studies by our group have identified several variants of phosphatidyl-ethanolamines bound the the SF-1 ligand-binding domain [42]. Crystal structures exist of SF-1 bound to phosphatidylglycerol [18] and phosphatidylcholine [19], while phosphatidic acid [18] and all phosphoinositides tested will bind directly and stoichiometrically to the purified SF-1 ligand-binding domain [18,43]. Data from our group suggests the signaling phosphoinositide PI(3,4,5)P3 is also an important regulatory ligand for SF-1 [16,43]. Studies published by other groups have suggested the complex of PI(3,4,5)P3 and SF-1 can be detected in cells using the PI(3,4,5)P3-specific PH-domain from BTK as a biosensor in fluorescence-recovery after bleaching (FRAP) experiments in human cells. The BTK-PH domain is well known to recognize PIP3 in membrane systems [50], these studies suggest that the siRNA-mediated knockdown of SF-1 decreases FRAP of fluorophore-tagged nuclear AKT to sites of laser-induced DNA damage [44]. Sphingolipids were also found associated with wild-type SF-1 by mass spectrometry, while several mutants of SF-1 designed to discourage phospholipid binding had significantly less sphingolipid associated with those mutant SF-1 proteins that had been purified from an adrenal cell line [45,46,51]. That PI(4,5)P2 was not identified in these experiments may be a function of how difficult it is to detect phosphoinositides by typical mass spectrometry-based lipidomics. Nevertheless, the data here and published elsewhere all suggest SF-1 likely binds a wide variety of phospholipids, including PI(4,5)P2, in human cells.

SF-1 acts in the same manner as a phosphoinositide transfer protein in vitro, extracting PI(4,5)P2 from micelles or vesicles without any input of energy from ATP, and without the aid of any other proteins in the test tube. Indeed, we took advantage of this activity when developing protocols to generate PI(4,5)P2 for crystallographic [16,19,52] and enzyme kinetic analyses [43]. This phospholipid transfer activity complicates the interpretation of any study that biochemically purifies SF-1 from cells as any phospholipids detected may have been acquired by the ability of SF-1 to extract phospholipids during biochemical purifcation. Our immunofluorescence data presented here suggest that in fixed human HEK cells SF-1 expression induces a nuclear signal cross-reactive with anti-PIP2 antibodies.

A major outstanding question in the field is how SF-1 initially acquires the PI(4,5)P2 phospholipid; e.g., is PI(4,5)P2 bound to SF-1 while SF-1 is being translated, or does SF-1 acquire phospholipids dynamically from cytoplasmic or nuclear membranes [53]? Another formal possibility is that SF-1 could acquire phospholipids via phospholipid exchange proteins such as PITP, although no exchange proteins are required for purified SF-1 to acquire phospholipids from bilayers or micelles in vitro. Little if any data have been published describing how SF-1 might acquire its stoichiometrically bound phospholipid in living cells [53], but there are examples in the literature of how other phospholipid-binding proteins and nuclear receptors do acquire phospholipids. In the case of Sec14/PITP, this occurs by entropy-driven phospholipid transfer from membranes [54] and can result in stimulation of phosphoinositide synthesis [55,56,57]. Studies of the sub-cellular location of specific transcript translation sites [58] have suggested translation by ribosomes on the ER does not require a classic signal sequence [59], and several transcripts encoding nuclear receptors have been discovered as physically associated with ribosomes on the ER [60]. No direct evidence suggests that SF-1 transcripts, in particular, are translated on the ER, but SF-1 has limited tissue and cell line expression, restricted to the gonads, adrenals, and one specific site within the hypothalamus (the ventral medial region) [61], making identification of any quantifiable SF-1 transcript unlikely when non-steroidogenic cell lines are used [60]. Thus, we cannot formally exclude that SF-1 acquires phospholipid ligands while SF-1 is being translated close to the ER [53]. Another possibility is that phospholipids are loaded into SF-1 without SF-1 interacting with a membrane, through the action of a phospholipid-transfer protein, although we are quick to add that no interactions of SF-1 with any known phosphoinositide transfer proteins have been reported to date. How SF-1 acquires phospholipids, including PI(4,5)P2, remains enigmatic, but the data presented here add to a growing body of structural, functional, and biochemical data suggesting a link between SF-1 and nuclear PI(4,5)P2 in cells. Further, that SF-1 is able to solubilize nuclear PIP2 to stabilize the hydrophobic acyl chains deep in the hydrophobic pocket of SF-1, provides one clear physicochemical format that can explain how non-membrane PIP2 can exist within the nucleoplasm, as suggested by the data generated by Lucio Cocco and Robin Irvine over 35 years ago [1].

## 5. Conclusions

We conclude induction of wild-type SF-1 in HEK cells results in a nuclear PIP2 antibody cross-reactive signal within 24 h of SF-1 induction. This signal is not induced by a pocket mutant of SF-1 that does not bind phospholipids, despite equal expression of the wild-type and pocket mutant proteins in these cells. The data further support a wide range of biochemical, biophysical, and structural studies, which all suggest PI(4,5)P2 is an endogenous regulatory ligand for SF-1 in human cells.

## Data Availability

Any and all data associated with this manuscript are available upon request to the corresponding author ray.blind@vanderbilt.edu, or via the website http://blindlab.org/protocols.

## Supplementary Materials

The following supplementary information can be downloaded at: https://www.mdpi.com/article/10.3390/biom13101509/s1. Figure S1: Triplicate, independent chambers of wild-type SF-1 HEK cells stained with anti-FLAG antibodies. Figure S2: Triplicates of independent chambers of wild-type SF-1 HEK cells stained with PI(4,5)P2 antibodies. Figure S3: Triplicates of independent chambers of wild-type SF-1 HEK cells stained with PIP3 antibodies. Figure S4: Triplicate images of independent chambers of pocket mutant SF-1 (A270W, L345F) HEK cells stained with PIP2 antibodies. Figure S5: Triplicate merged IF images (DAPI (blue) merged with PIP2-antibody (red)) to directly compare PIP2-anti-body staining between wild type SF-1 (WT, on left of each panel) vs. pocket mutant SF-1 (PM, A270W, L345F, on right of each panel).
