# Supplementary material for: SF-1 Induces Nuclear PIP2
Source: Biomolecules. 2023 Oct 12;13(10):1509. doi: 10.3390/biom13101509 (PMC10604688; doi:10.3390/biom13101509)
Supplement: Supplementary file 1 [file biomolecules-13-01509-s001.zip › biomolecules-2615405-supplementary.pdf]

Supplemental Materials.
SF-1 induces nuclear PIP2.

Ethan S. Chi[1], Elizabeth A. Stivison[1] and Raymond D. Blind[1,2]

[1]Department of Medicine, Division of Diabetes, Endocrinology and Metabolism, Vanderbilt University Medical Center, Nashville, TN 37232

[2]To whom correspondence should be addressed: ray.blind@vanderbilt.edu

Supplemental Figure S1, Chi, et al.

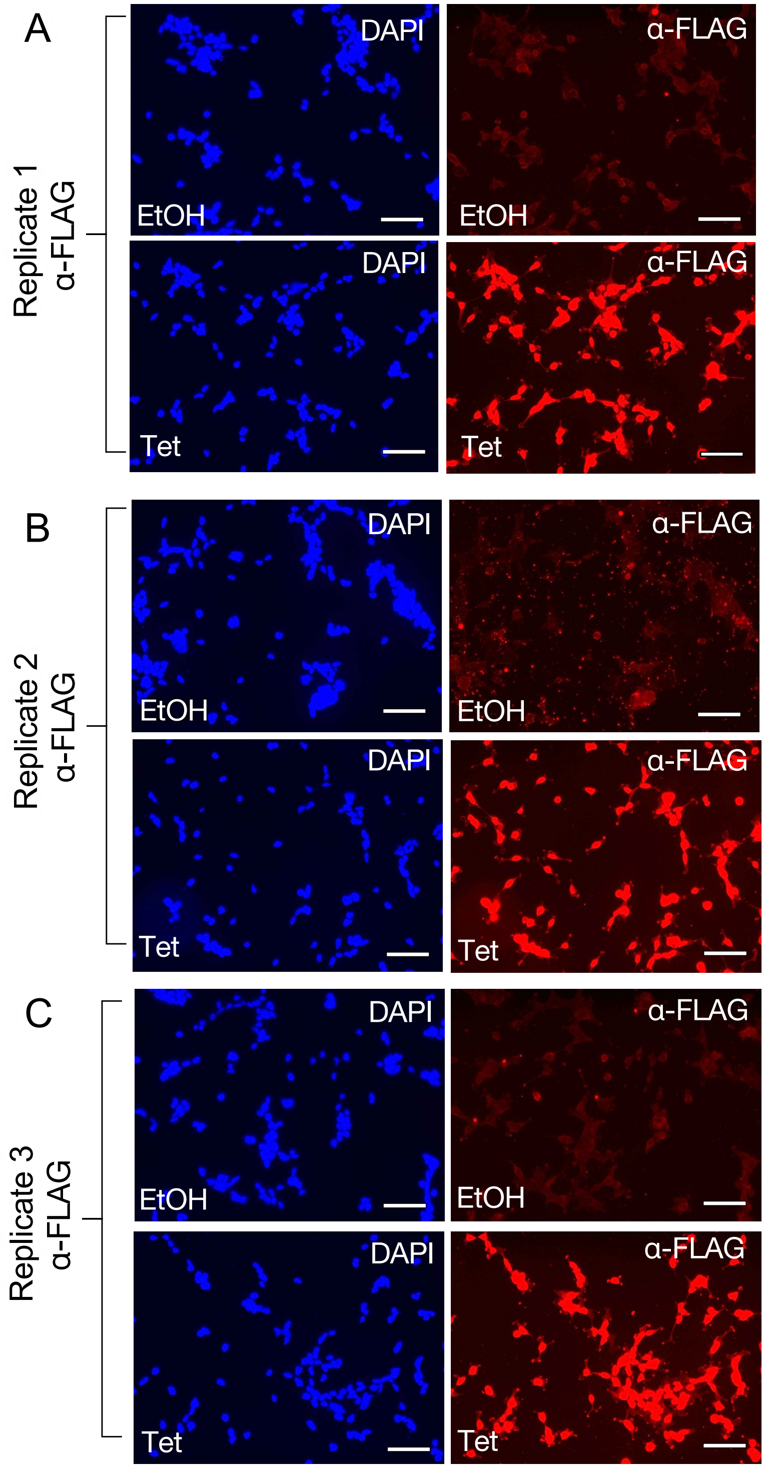

Supplemental Figure S1. *Triplicate, independent chambers of wild-type SF-1 HEK cells stained with anti-FLAG antibodies*. HEK cells bearing a stable, tetracycline (Tet) inducible wild type SF-1 were stained for **A.** Replicate 1 **B**. Replicate 2 or **C**. Replicate 3 with DAPI (left) and α-FLAG antibodies and IF images collected. Scale bar represents 125um, images were collected at 40X with identical settings, all images had brightness increased by 50%. These data confirm SF-1 is expressed in the nucleus of these cells, as observed in previous publications.

Supplemental Figure S2, Chi, et al.

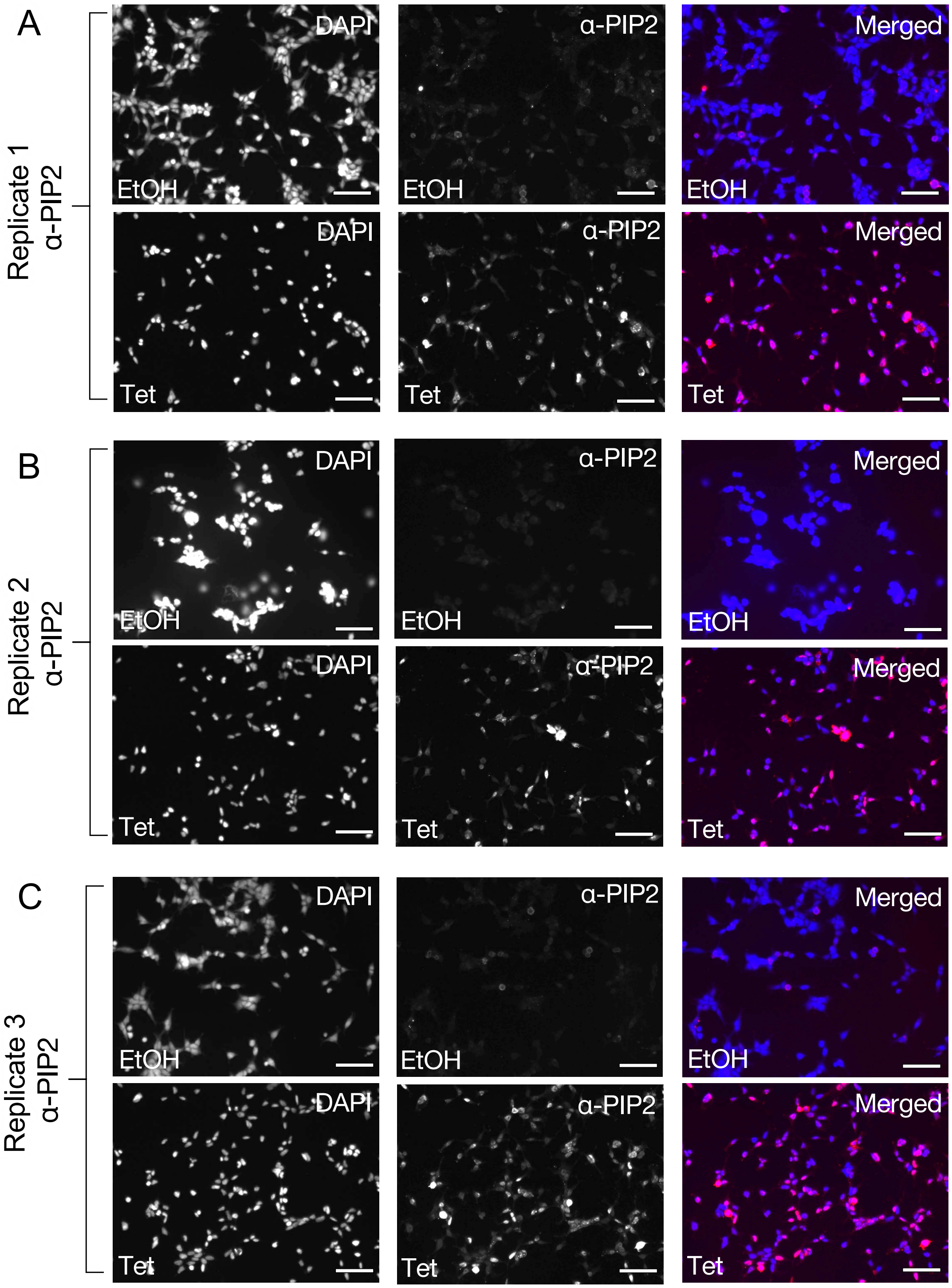

Supplemental Figure S2. *Triplicates of independent chambers of wild-type SF-1 HEK cells stained with PI(4,5)P2 antibodies*. HEK cells bearing a stable, tetracycline (tet) inducible wild type SF-1 were stained for **A.** Replicate 1 **B**. Replicate 2 or **C**. Replicate 3 with DAPI (left) and α-PIP2 (middle) and IF images collected. Scale bar represents 125um, images were collected at 40X with identical settings. All images had brightness increased by 50%, DAPI (blue) and anti-PIP2 (red) image color was desaturated in the non-merged images.

Supplemental Figure S3, Chi, et al.

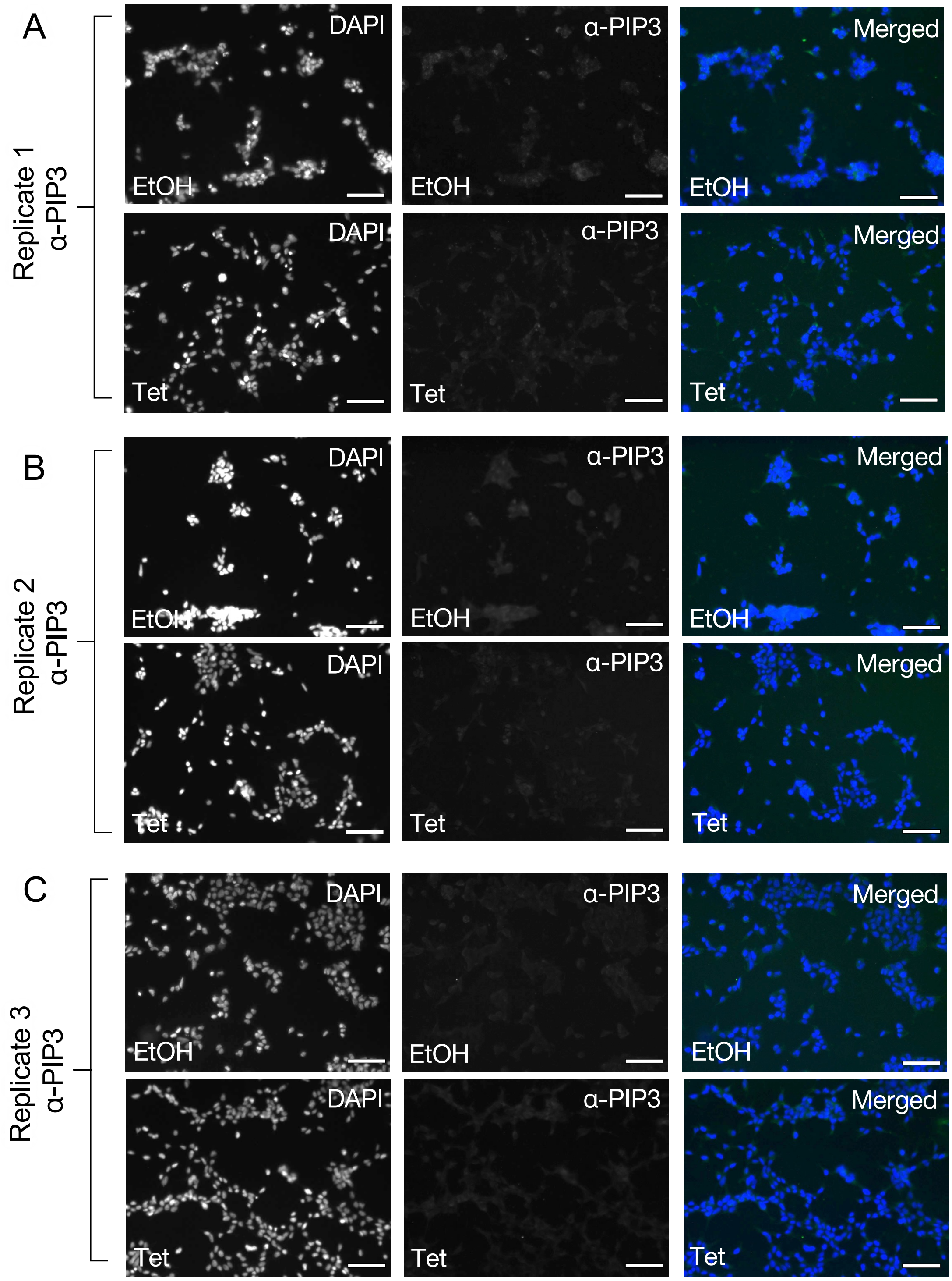

Supplemental Figure S3. *Triplicates of independent chambers of wild-type SF-1 HEK cells stained with PIP3 antibodies*. HEK cells bearing a stable, tetracycline (Tet) inducible wild type SF-1 were stained for **A.** Replicate 1 **B**. Replicate 2 or **C**. Replicate 3 with DAPI (left, blue) and α-PIP3 (middle, green) and IF images collected. Scale bar represents 125um, images were collected at 40X with identical settings. All images had brightness increased by 50%, DAPI (blue) and anti-PIP3 (green) image color was desaturated in the non-merged images.

Supplemental Figure S4, Chi, et al.

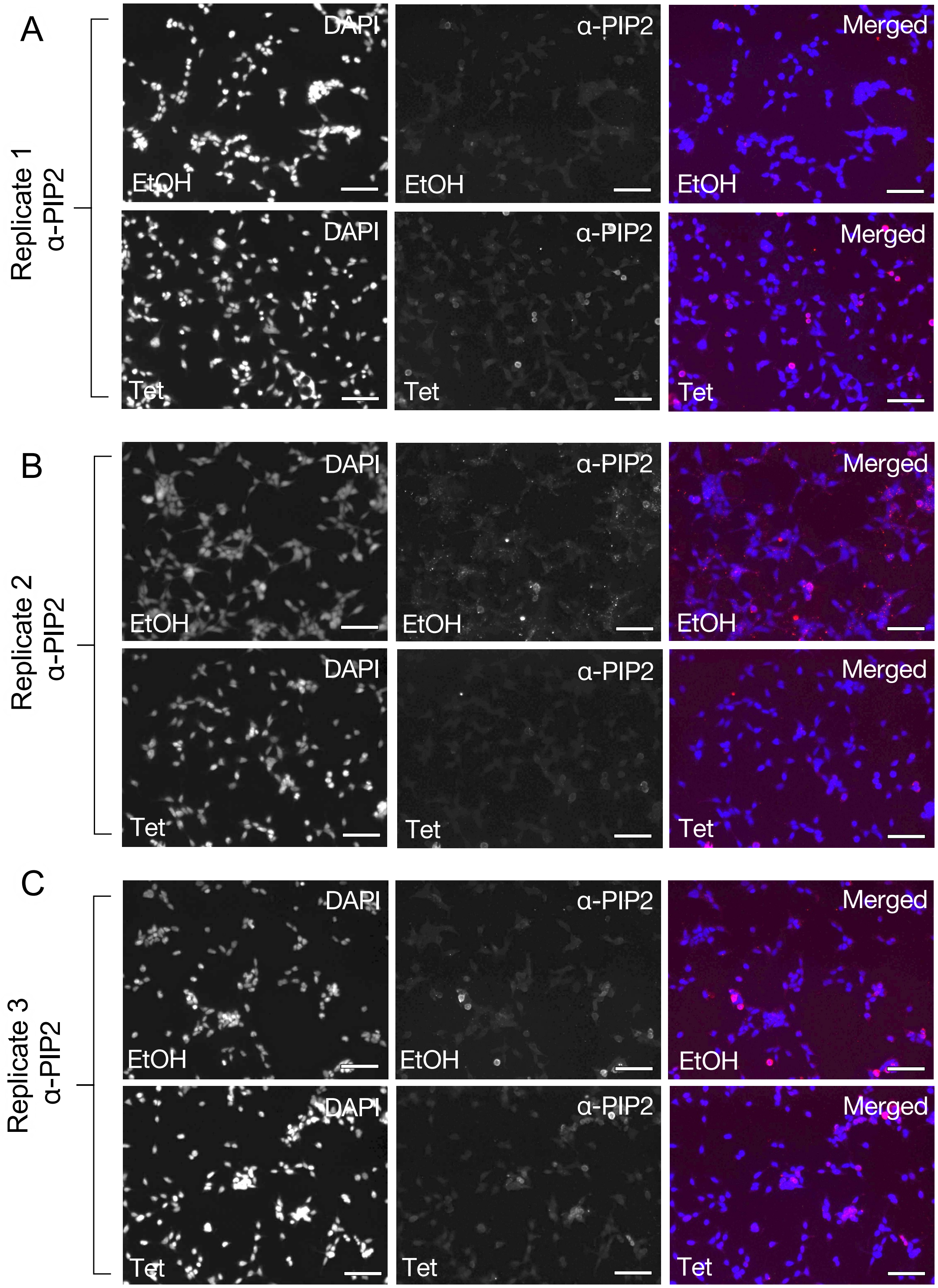

Supplemental Figure S4. *Triplicate images of independent chambers of* ***pocket mutant*** *SF-1 (*A270W, L345F) *HEK cells stained with PIP2 antibodies*. HEK cells bearing a stable, tetracycline (Tet) inducible pocket mutant SF-1 (A270W, L345F) stained for **A.** Replicate 1 **B**. Replicate 2 or **C**. Replicate 3 with DAPI (left) and α-PIP2 (middle) and IF images collected. Scale bar represents 125um, images were collected at 40X with identical settings. All images had brightness increased identically by 70% so that low levels of PIP2 antibody staining could be visualized, DAPI (blue) and anti-PIP2 (red) image color was desaturated in the non-merged images.

Supplemental Figure S5, Chi, et al.

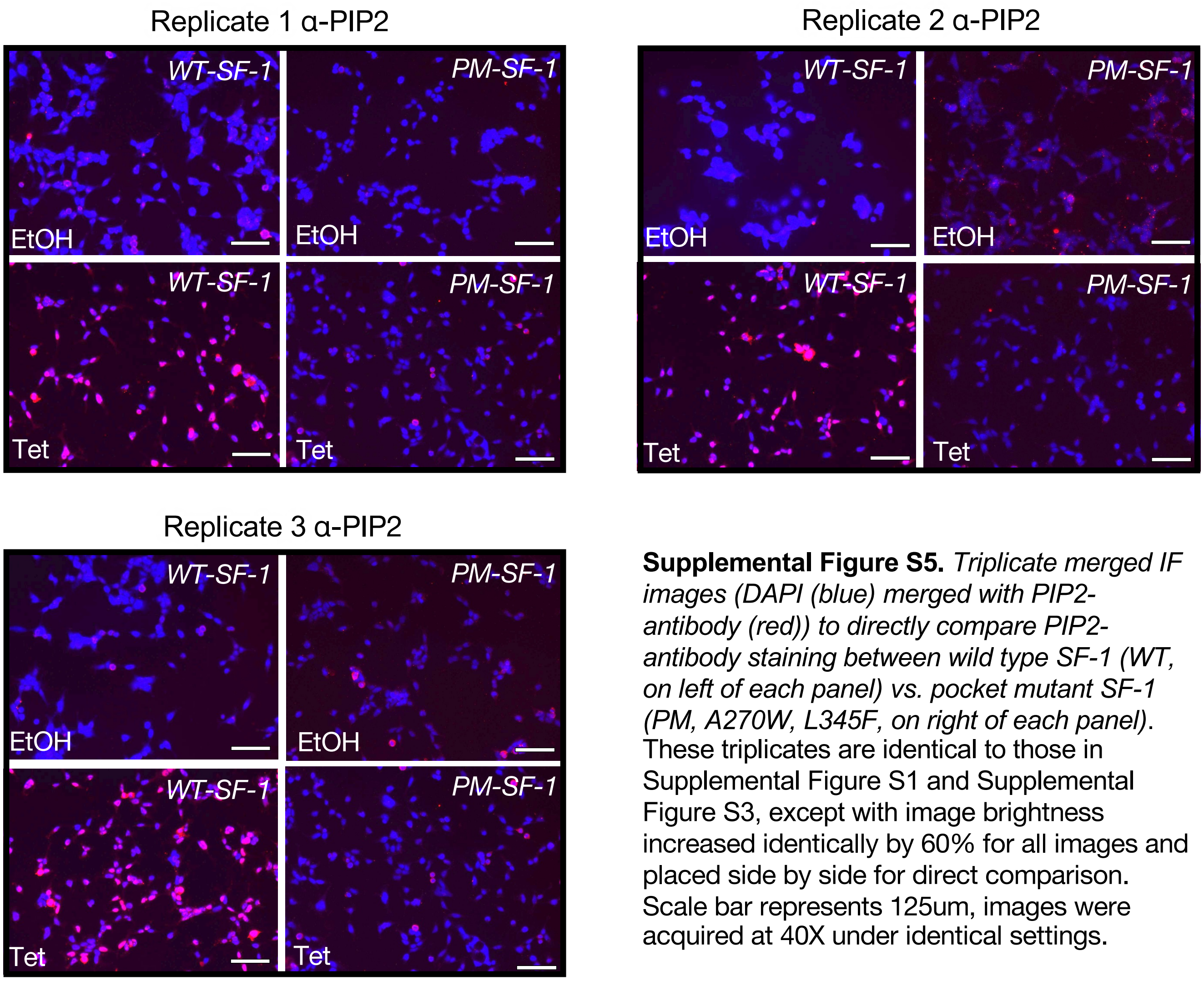

Supplemental Figure S5. *Triplicate merged IF images (DAPI (blue) merged with PIP2-antibody (red)) to directly compare PIP2-antibody staining between wild type SF-1 (WT, on left of each panel) vs. pocket mutant SF-1 (PM, A270W, L345F, on right of each panel)*. These triplicates are identical to those in Supplemental Figure S1 and Supplemental Figure S3, except with image brightness increased identically by 60% for all images and placed side by side for direct comparison. Scale bar represents 125um, images were acquired at 40X under identical settings.
