# Supplementary figures and images for: SF-1 Induces Nuclear PIP2
Source: Biomolecules. 2023 Oct 12;13(10):1509. doi: 10.3390/biom13101509 (PMC10604688; doi:10.3390/biom13101509)

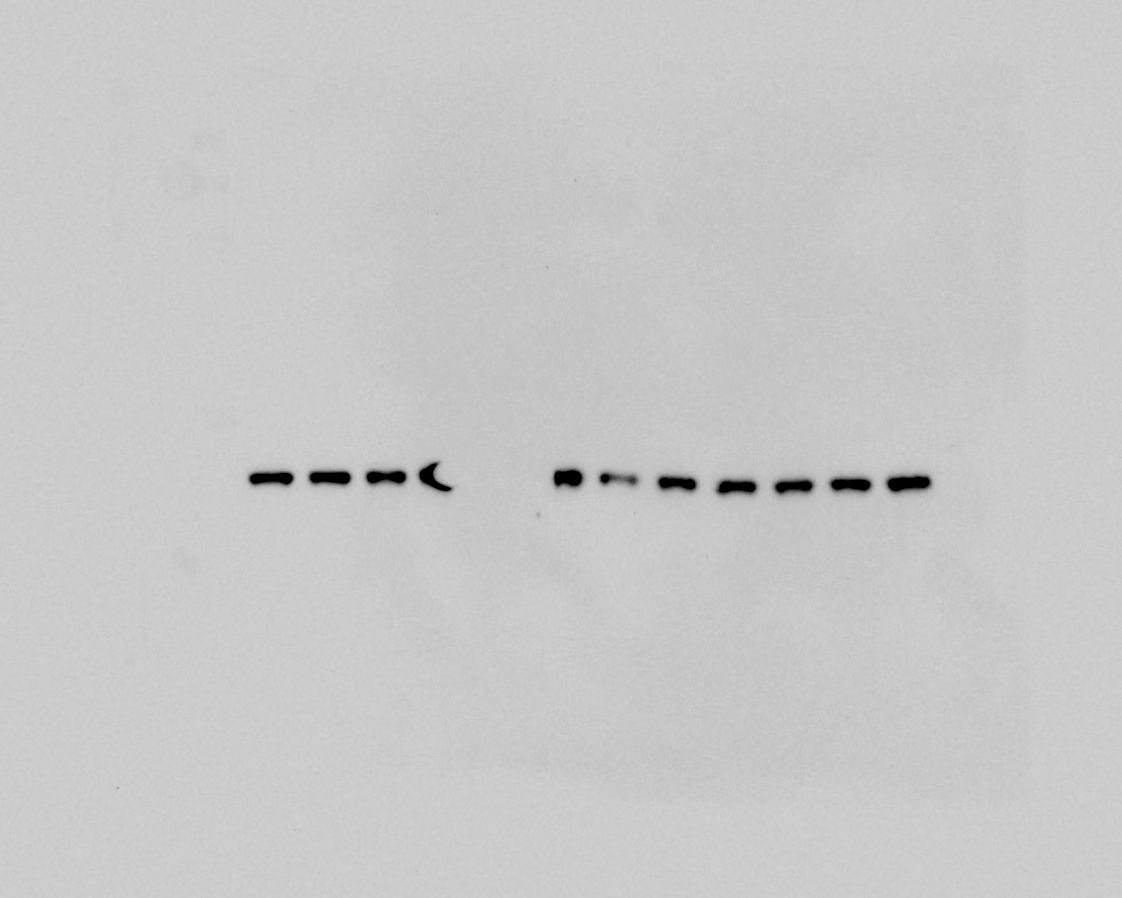

Supplement: Supplementary file 1 [file biomolecules-13-01509-s001.zip › biomolecules-2615405-All_ACTIN_Westerns.tif]

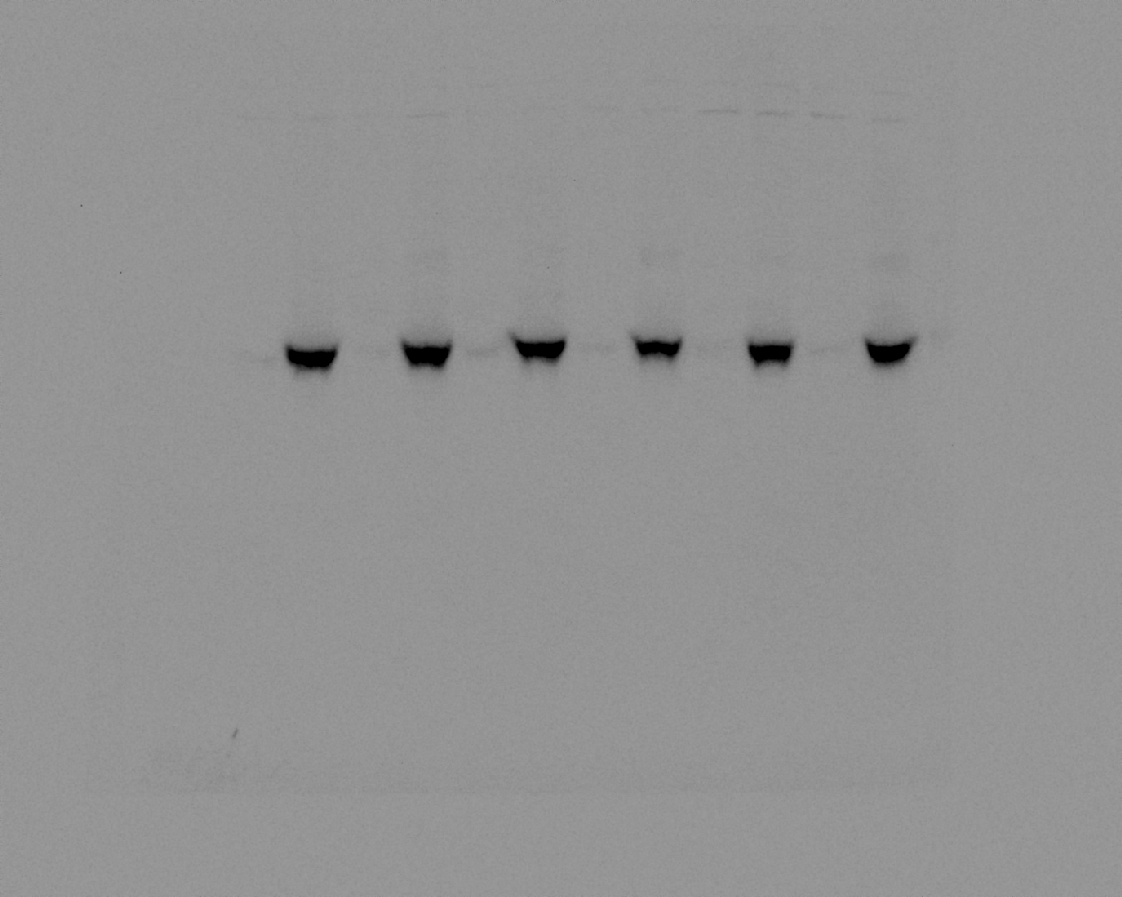

Supplement: Supplementary file 1 [file biomolecules-13-01509-s001.zip › biomolecules-2615405-All_FLAG_Westerns.tif]
